# Categorization of Emotional Faces in Insomnia Disorder

**DOI:** 10.3389/fneur.2020.00569

**Published:** 2020-06-19

**Authors:** Song Xu, Xueping Liu, Lun Zhao

**Affiliations:** ^1^Department of Senile Neurology, Shandong Provincial Hospital, Cheeloo College of Medicine, Shandong University, Jinan, China; ^2^Anti-Aging Monitoring Laboratory, Shandong Provincial Hospital, Cheeloo College of Medicine, Shandong University, Jinan, China; ^3^Department of Anti-Aging, Shandong Provincial Hospital, Cheeloo College of Medicine, Shandong University, Jinan, China; ^4^Department of Senile Neurology, Shandong Provincial Hospital Affiliated to Shandong First Medical University, Jinan, China; ^5^School of Educational Science & Technology, Liaocheng University, Liaocheng, China; ^6^Bell Laboratory of Brain Science, Shandong Kang Rida Life Sciences Research Co. Ltd, Jinan, China

**Keywords:** insomnia, facial expression, positive classification advantage, configural processing, reaction times

## Abstract

It has been proved that emotionally positive facial expressions are categorized much faster than emotionally negative facial expressions, the positive classification advantage (PCA). In the present study, we investigated the PCA in primary insomnia patients. In comparison with controls, insomnia patients categorized emotional faces more slowly but there was no significant reduction in accuracy. In normal controls, happy faces were categorized faster than sad faces (i.e., PCA), which disappeared in the inverted condition. Insomnia patients did not show evident PCA except for the overall delayed response for the inverted compared to the upright condition. These data suggest the dysfunction of categorization of emotional faces in insomnia patients.

## Introduction

Insomnia is characterized as dissatisfaction with sleep quantity or quality and is associated with difficulty initiating or maintaining sleep and early-morning waking with inability to return to sleep (1, 2). Despite adequate opportunity for sleep, these nocturnal symptoms occur and are accompanied by clinically significant distress or impairment of daytime functioning, including fatigue, decreased energy, poor cognitive functions, mood disturbances, and distress or interference with personal functioning (3, 4), which become increasingly serious as the degree of insomnia increases (5). The criteria specify that symptoms must cause clinically significant functional distress or impairment; be present for at least 3 nights per week for at least 3 months; and not be linked to other sleep, medical, or mental disorders (1). According to the DSM-IV standard, the prevalence rate of insomnia worldwide is estimated to be about 10% in the population meeting the diagnostic criteria (6–8). Primary insomnia is associated with decreased quality of life and reduced work efficiency (9), and it also increases the incidence of mental illness, accidental injuries, and occupation of medical resources, which is associated with significant direct and indirect costs (9–13).

Considerable attention has been made to investigate fatigue, work productivity, and cognitive performance, and impair psychosocial functioning in the research literature (14–16). Interestingly, several preliminary work reveals that the impairment in social interactions and emotion regulation is a salient concern for patients with insomnia (17). To date, however, there is relatively little investigation of how insomnia affects emotional processing. Facial expressions play an important role in social life. Facial expressions represent a vital role in social life. The information is important for interpreting how others feel and their behavioral tendencies. Some efforts have been made to investigate the processes of facial expressions in patients with sleep disorder. For example, using schematic images facial recognition, was impaired in the sleep-deprived state (18, 19). Similarly, Kyle et al. (20) found that chronic insomnia is associated with reduced ratings of emotion intensity for face expressions displaying sadness and fear, using a facial expression recognition task. Moreover, Tatjana et al. (21) indicated that both patients with psycho-physiologic insomnia and patients with sleep apnea showed significantly lower performance in the FEEL test as compared to the control group.

There is consistent evidence that positive (happy) facial expressions are recognized substantially faster than negative facial expressions (sad, anger, disgust) (22–26), the phenomenon of positive classification advantage (PCA). It should be noted that the task of simple two-choice classification of PCA actually reflects a unique mechanism from recognition and memory of facial expressions in most previous studies. In general, facial classification is based on visual information that is similar to all “facial action patterns,” which is one key form of expert visual processing. Regardless of the face that makes up the face, the facial expression classification process is based on the extraction of facial expression attributes (27). To our knowledge, no study directly investigated the PCA in patients with insomnia, which would be explored using schematic faces with upright and inverted conditions.

It is widely accepted that adults' expertise in recognizing faces is attributed to configural processing (28). There are three types of configural processing: (1) face featural information—sensitivity to first-order relations—seeing a stimulus as a face because its features are arranged with two eyes above a nose, which is above a mouth; (2) holistic processing—gluing together the features into a gestalt; and (3) sensitivity to second-order relations—perceiving the distances among features. Inversion of a face interferes with all three types of configural processing (28–30). Recently, there was evidence that the face of orientation change has different influences on configural processing, showing that the performance was impaired most conspicuously for faces rotated 90 and 120° compared with the upright condition, indicating that the change in face orientation makes configural processing less efficient (31).

It has been shown that the configural analysis underlying face recognition is also applicable to facial-emotion recognition as it depends on facial features and spatial arrays (32, 33). For example, McKelvie and his colleagues evaluated the effect of face inversion on facial-emotion recognition and found that inversion impaired the recognition of sadness, fear, angry, and disgusted, but not of happy expressions (33). Recently, there was evidence that the PCA of the inverted faces disappeared, indicating that one of the PCA sources is the configural analysis (reflected by face inversion), which is applied by default while categorizing facial emotions (34).

The present study was designed to directly test the configural processing of emotional faces in insomnia patients by examining the face-inversion effect in the emotional categorization task. It has been shown that even schematic faces triggered off the face-sensitive N170 (35). In addition, Wright et al. found that schematic faces also significantly increased fMRI signals, which were found in the amygdala, hippocampus, and prefrontal cortex, suggesting that schematic faces are reliable and useful for investigating brain responses to emotional faces due to their simplicity (36). In the current study, schematic emotional faces were used to minimize the variance associated with genuine facial photographs. A series of schematic emotional faces was presented in both upright and inverted conditions.

## Methods

### Participants

We recruited 30 primary insomnia patients (age 25–55 years) and 30 age- and education-matched healthy controls. All patients (see the following paragraphs for specific inclusion criteria) were from outpatients due to insomnia who visited the neurology clinic, Shandong Provincial Hospital in Jinan, China. The healthy volunteers had no history of any major psychiatric disorders or major physical illnesses and were not taking any medication that affects the central nervous system. All participants reported that they had normal or corrected-to-normal vision and gave written informed consent prior to participation in the study. This study was approved by the Ethics Committee of Shandong Provincial Hospital, which acts to meet the demands of the Helsinki Declaration.

In order to exclude the influence of testing time (i.e., morning, afternoon, and evening) on patients and controls, we chose a fixed time period between 18:00 and 19:00 to conduct this study (20).

### Sleep Status

Diagnosis was validated according to the ICSD-3 (2) criteria and a polysomnography (PSG) to exclude the presence of sleep apnea or periodic leg movements in sleep. Inclusion criteria were as follows: (1) report of sleep disturbance for at least 3 nights per week for ≥6 months; (2) evidence of conditioned sleep difficulty and/or heightened arousal in bed—sleep onset latency (SOL) and/or wake time after sleep onset (WASO) > 30 min/total sleep time (TST) ≤ 6 h/sleep efficiency (SE) <85%; (3) daytime impairment attributed to disturbed sleep; (4) insomnia was not due to any other current primary sleep, medical, or psychiatric disorder; (5) affective disorders, especially depression, were excluded in a structured interview conducted by an experienced psychiatrist; (6) any severe neurological or other medical condition led to the exclusion of the patients; (7) right-handed (Edinburgh Handedness Inventory, EHI) (37). Exclusion criteria based on PSG for patients were apnea–hypopnea index (AHI) > 5 and/or periodic limb movements of sleep (PLMS) arousal index > 15/h. In addition, hypnotic drugs were discontinued 1 week before the test for insomnia patients.

### Polysomnography

The PSG signals for all subjects were evaluated for one night with a standard PSG montage in the sleep laboratory. The following sleep parameters were recorded: SOL, WASO, TST, time in bed (TIB), and SE. The PSG measurements are presented in Table 1.

**Table 1:** Demographic and clinical data of participants.

**Characteristics**	**Healthy controls (*n* = 30) (mean ± SD)**	**Insomnia (*n* = 30) (mean ± SD)**
Age, years	37.65 ± 8.85	39.77 ± 7.38
Education, years	15.00 ± 2.63	13.83 ± 2.63
SCI, score	27.06 ± 1.90	12.50 ± 1.97^*^
SDS, score	36.13 ± 3.33	37.77 ± 3.64
SOL (min)	8.0 ± 6.9	39.1 ± 22.4^*^
WASO (min)	8.2 ± 10.2	65.2 ± 46.7^*^
TST (min)	432.9 ± 31.6	321.5 ± 50.2^*^
TIB (min)	498.1 ± 58.0	500.6 ± 60.1
SE (%)	90.1 ± 5.2	68.1 ± 11.2^*^
AHI	3.1 ± 3.2	2.8 ± 3.0
PLMS index	2.1 ± 1.0	1.9 ± 1.6

*SCI, Sleep condition indicator; SDS, Self-rating depression scale; SOL, Sleep onset latency; WASO, Wake time after sleep onset; TST, Total sleep time; TIB, Time in bed; SE, Sleep efficiency; AHI, Apnea hypopnea index; PLMS index, Periodic limb movements of sleep arousal index*.

* *p < 0.05 for group comparison*.

### Sleep Condition Indicator

A validated Chinese version of the SCI was used to assess the participants' self-reported insomnia symptoms (38). The SCI asks eight items concerning an individual's sleep condition during the recent month in a 0–4 Likert style, with an item score ≤ 2 considered as an item-wise insomnia symptom and an item score ≥ 3 as no insomnia symptom.

The Chinese SCI has been validated and recommended as a screening tool for clinical insomnia with an original insomnia/non-insomnia cutoff at 21/22 (39). The short-form SCI contains two most important items indexing insomnia symptoms (item 3: poor sleep nights per week, and item 7: extent troubled by sleep problems) in the 8-item SCI (38). In the present study, according to the SCI item score threshold criteria for insomnia disorder (score ≤ 2), the results for participants are shown in Table 1.

### Stimuli

**Figure 1:**
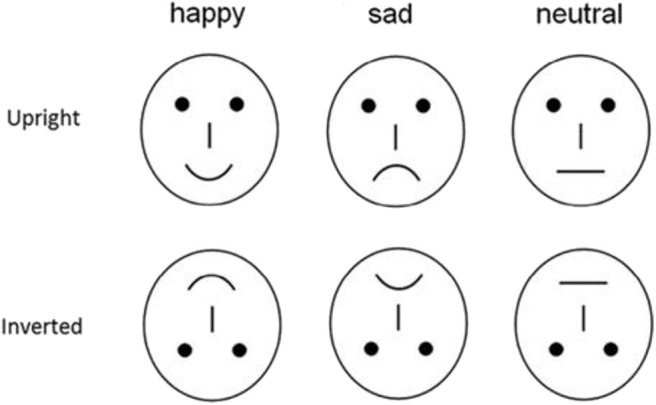
Schematic stimuli used in the present study.

To avoid the low-level processing of facial features as well as boredom by the excessive repetition of one single model, each emotional category consisted of 20 different schematic face models by manipulating the distance among facial features and by manipulating the shape of the facial features (Figure 1) (24, 34). All stimuli were presented at the center of a video monitor and viewed from a distance of 100 cm at a visual angle of ~7.27 ^*^ 6.06°.

### Procedure

The participants were seated in a dimly lit and sound-attenuated cabin and instructed to classify each face by the expression it represented and to respond to sad, happy, and neutral faces by pressing correspondingly labeled buttons on the keyboard with the left index finger or right index finger, respectively. Speed and accuracy were equally emphasized. All 480 stimuli were randomly presented in a mixed design, with four blocks of 120 stimuli each, with a short break in between, and the labels of the response buttons (happy, sad, neutral) were counterbalanced across the participants. Each face was presented for 300 ms with an inter-trial interval ranging randomly between 600 and 800 ms, starting after response. The participants completed one practice sequence of 30 stimuli (five from each type, equally representing the three facial expressions). These stimuli were not used in the main experiment.

### Statistical Analysis

Statistical analysis was performed using SPSS 20.0 (Chicago, IL, USA) and the chi-square test was used to compare numerical data. Differences in continuous demographic and clinical data and scale scores were analyzed by independent-samples *t*-tests. Reaction times (RTs) (from the stimulus onset) and accuracy rates were recorded and analyzed using a three-way analysis of variance (ANOVA) with Expression (happy and sad) and Orientation (upright, inverted) as within-subject factors and Group (normal controls, patients) as a between-subject factor.

## Results

### Accuracy Rates

**Figure 2:**
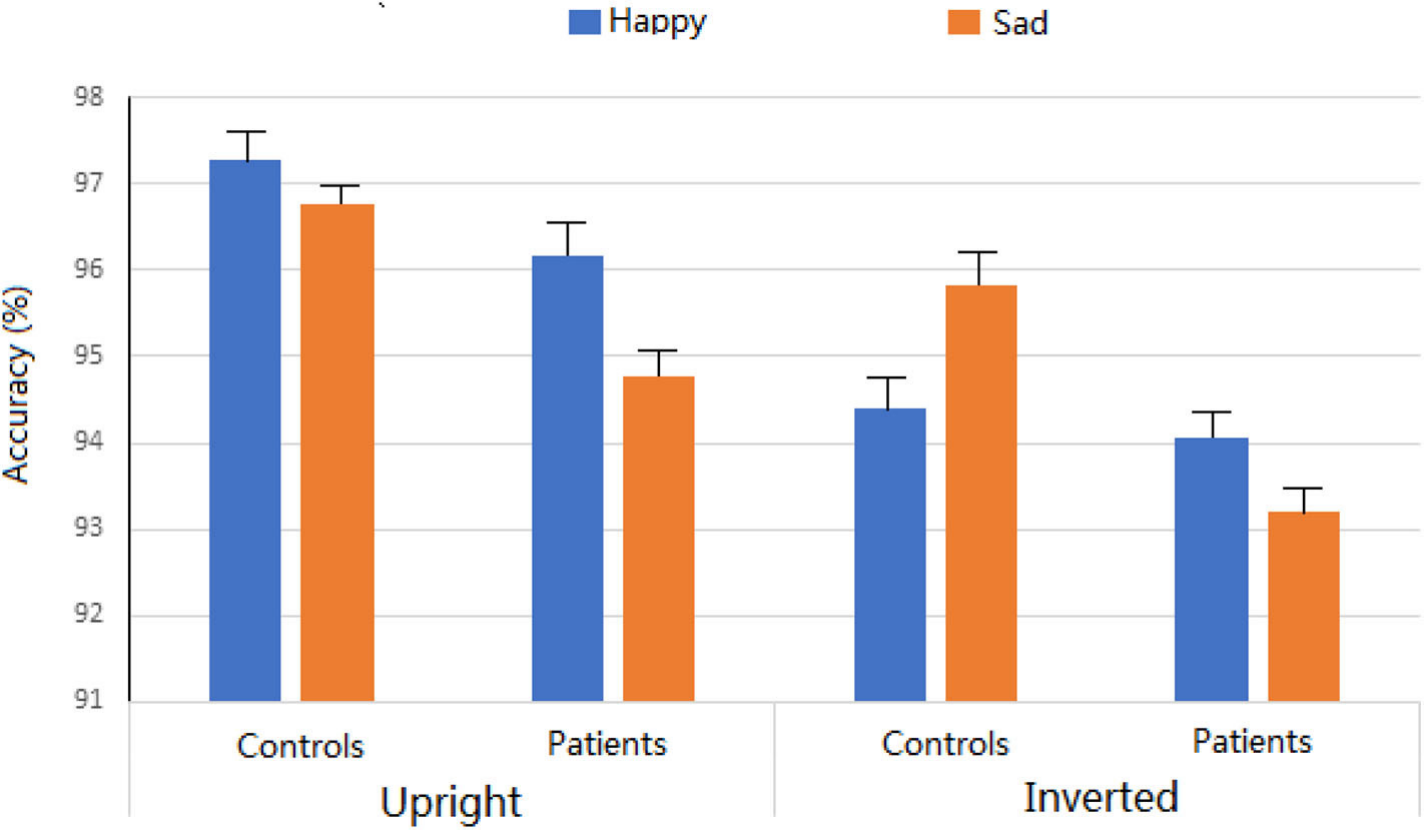
Accuracy rates with standard deviation for different expressions in two groups.

The three-way ANOVA was conducted for the percentage of correct responses. Across experimental conditions, there was no significant difference between insomnia patients (94.55%) and controls (96.07%). The main effects of Expression were not significant (95.5% and 95.1 for happy and sad faces, respectively; *F* < 1). The main effect of Orientation was significant, *F* = 18.6, *p* < 0.001, partial η^2^ = 0.239, showing more accuracy for upright (96.2%) than for inverted (94.4%) conditions. Interestingly, the two-way interaction of Expression ^*^ Orientation was significant, revealing that the inversion effect was evident for happy faces (*p* < 0.001) and not for sad faces (*p* = 0.065). No other main effects and interactions reached significant level (*p*s > 0.1) (Figure 2).

### Reaction Times

For each participant, incorrect responses or responses with RTs more than ±2 SDs from the mean in each condition were excluded for RT analysis. The RTs were analyzed using the same statistical model as that for percentages of correct responses. Overall, the patients exhibited slowly response speed (706.8 ms) than the controls (651.6 ms; *F* = 7.6, *p* < 0.01, partial η^2^ = 0.114). Across groups, face inversion modulated the facial classification by expressions, *F* = 91.0, *p* < 0.001, partial η^2^ = 0.607, showing the faster response for upright (648.7 ms) than inverted (709.7 ms) conditions. The main effect of Expression was also significant, *F* = 15.3, *p* < 0.001, partial η^2^ = 0.206, revealing that happy faces (668.6 ms) were categorized by expressions more quickly than sad faces (689.8 ms) (i.e., PCA).

**Figure 3:**
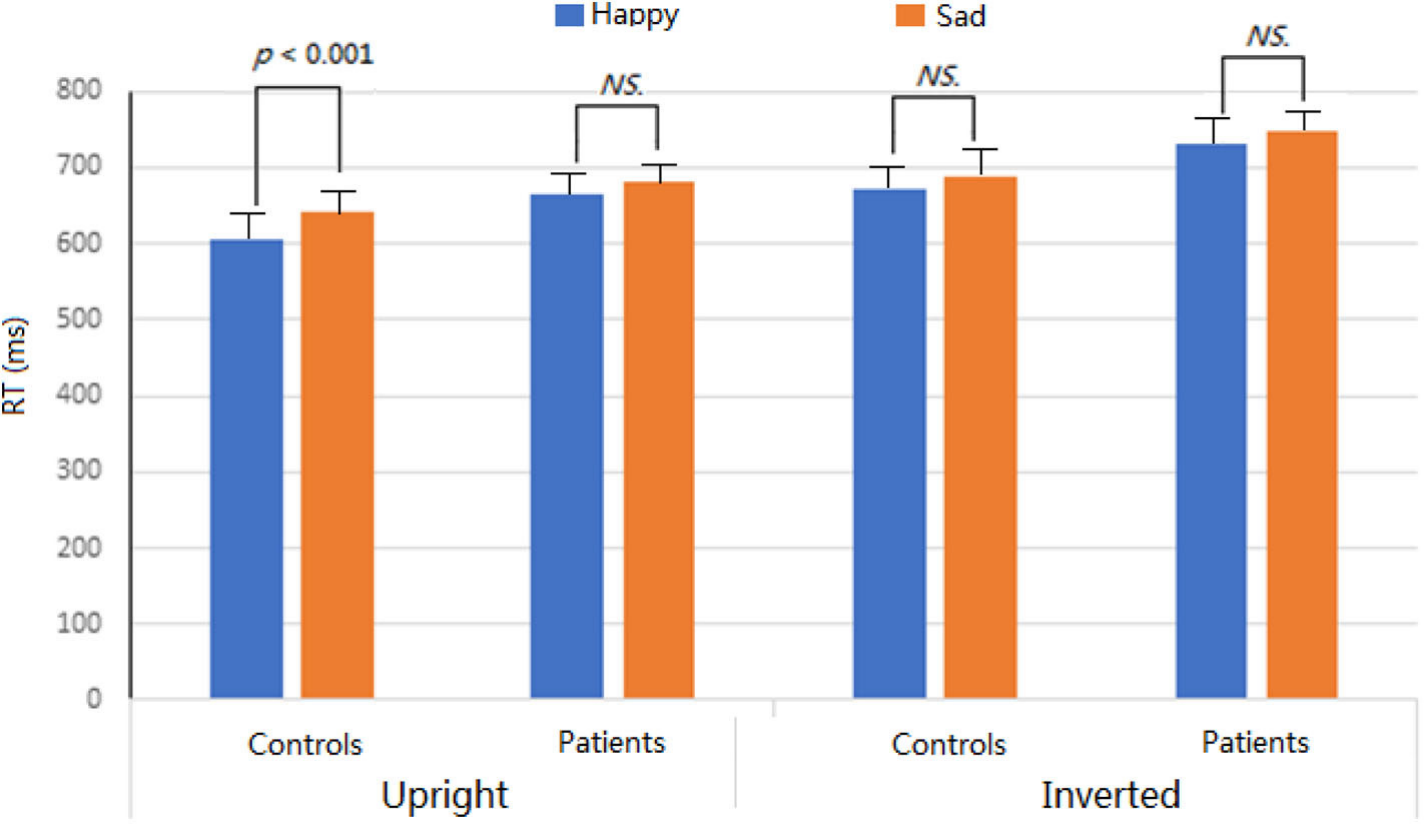
RTs with standard deviation for different expressions in two groups.

We found a significant interactions of Group ^*^ Expression ^*^ Orientation (*p* < 0.05), which was further analyzed for two groups, respectively. In controls, the main effect of Expression was significant, *F* = 11.9, *p* < 0.001, partial η^2^ = 0.167, revealing that happy faces (638.5 ms) were categorized by expressions more quickly than sad faces (664.7 ms) (i.e., PCA). This effect was also modulated by facial inversion, *F* = 37.6, *p* < 0.001, partial η^2^ = 0.389, showing that the PCA was evident for the upright condition (*p* < 0.001) but not for the inverted condition (*p* = 0.069) and that facial inversion delayed the response speed for both happy (*p* < 0.001) and sad (*p* < 0.001) faces. In patients, only the main effect of Orientation was significant, *F* = 54.07, *p* < 0.001, partial η^2^ = 0.478, showing the faster response for upright (648.7 ms) than for inverted (709.7 ms) conditions. No other main effects and interactions were significant (*p*s > 0.05) (Figure 3).

## Discussion

To our knowledge, very few studies have directly compared facial classifications by expressions between insomnia patients and controls. Although accuracy rates were similar for two groups, we found significant group differences of RTs. In the normal control group, the happy face was classified faster than sad faces (i.e., PCA), which disappeared in the inverted condition. Compared to the control group, patients with insomnia evaluated the emotional faces more slowly and did not show significant PCA.

Our findings in controls are consistent with previous studies showing the classification advantage for happy vs. sad faces, i.e., PCA (23, 24). In addition, it was well-known that the effect of face inversion related to the fact that the recognition is seriously impaired for inverted relative to upright faces. Because the configural processing in this study was task-irrelevant, the fact that PCA disappeared in inversion condition implicated that configural analysis was one of the causes of PCA used by default in classifying facial emotions. The fact that the insomnia patients did not show evident PCA with generally delayed response speed, regardless of the condition (upright or inverted), suggests the dysfunction of categorization of expressional information in insomnia patients (17–19). In previous studies, biased interpretation of emotional information after insomnia has typically been ascribed to impaired function of limbic structures, such as anterior cingulate cortex and amygdala and the functional connectivity between these limbic structures toward emotional stimuli and the prefrontal cortex (40, 41).

The present insomnia patients categorized emotional faces more slowly and did not show evident PCA, implicating the dysfunction of processing emotional faces in insomnia patients. Some research has accumulated, suggesting a robust impairment in emotion recognition in insomnia, especially regarding facial emotion recognition (42). Kyle et al. (20) reported that patients with insomnia did not differ from normal controls with respect to categorization of facial expressions, but the subjective intensity ratings of fear and sadness were blunted in insomnia and that chronic insomnia was associated with reduced ratings of emotion intensity for face expressions displaying sadness and fear for the first time and emotional intensity judgments were not found to correlate with sleep diary responses, measures of sleepiness, or daytime functioning. Tatjana et al. found that compared to the control group, both patients with psychophysiologic insomnia and sleep apnea showed significantly lower performance in the FEEL test, requiring participants to categorize and rate the intensity of six emotional expression categories (anger, anxiety, fear, happiness, disgust, and sadness) and differences were seen in the scales happiness and sadness (21). Although the present study is the first to investigate the face classification by emotion in insomnia patients, the disappeared PCA was indeed based on the slower response to happy faces vs. sad faces, that is, the more attentional resources for categorizing a face as happy emotion in patients, indicating the deficit of processing happy faces in insomnia patients.

Actually, it is well-known that insomniacs have difficulties in the emotion regulation due to the hyper-arousal during sleeping (17, 43). For example, Wassing et al. (43) found that the overnight resolution of distress from shame is compromised in people with insomnia, and this deficit contributes to hyper-arousal, revealing important associations between key characteristics of both insomnia and depression. The present dysfunction of categorization of expressional information could be relevant to the dysfunction of emotion regulation in insomnia. However, in our recent work (Zhao et al., unpublished data), we did not find the dysfunction of the PCA in depressive patients although the overall RTs were slower for depression due to the general cognition dysfunction. As mentioned in the recent review paper (17) and the above discussion, in addition to emotional regulation, lack of sleep significantly influences emotional processing at many aspects, such as emotional reactivity, emotional memory, empathic behavior, etc. Although the relationship between categorizing emotional faces and the chronic insomnia need further investigation, the present study extends the previous report especially for simple emotional classification.

Overall, the present study investigated whether there is a dysfunction of categorizing emotional faces in patients with insomnia. In comparison with controls, insomnia patients categorized emotional faces more slowly but with similar accuracy. Analyzing the RTs, we found that, in normal controls, happy faces were categorized more quickly than sad faces (i.e., PCA), which disappeared in the inverted condition, while the insomnia patients did not show evident PCA. These data further suggest the dysfunction of categorization of expressional information in insomnia patients and reveal important associations between insomnia and emotional processing. Future studies should further clarify the specificity of sleep-dependent emotional processing, which is an important issue for significant implications for clinical settings, e.g., achieving better understanding and treatment of affective or anxiety disturbances in patients with disturbed sleep.

## Data Availability Statement

The raw data supporting the conclusions of this article will be made available by the authors, without undue reservation, to any qualified researcher.

## Ethics Statement

The studies involving human participants were reviewed and approved by participants gave written informed consent prior to participation in the study, and the project was approved by the Ethics Committee of Shandong Provincial Hospital, which acts to meet the demands of the Helsinki Declaration. The patients/participants provided their written informed consent to participate in this study.

## Author Contributions

SX finished the data collection and the draft. XL and LZ finished the design and revised the manuscript. All authors contributed to the article and approved the submitted version.

## Conflict of Interest

LZ was employed by company Bell Laboratory of Brain Science, Shandong Kang Rida Life Sciences Research Co. Ltd, Jinan, China. The remaining authors declare that the research was conducted in the absence of any commercial or financial relationships that could be construed as a potential conflict of interest.
